# Supplementary material for: Causal interplay between lactose intolerance and gut microbiota: a combined bidirectional Mendelian randomization and in vivo validation study
Source: Front Nutr. 2026 Jun 1;13:1803337. doi: 10.3389/fnut.2026.1803337 (PMC13265576; doi:10.3389/fnut.2026.1803337)
Supplement: Supplementary file 1 [file Data_Sheet_1.zip › supplementary materials/Forward/leave one out cross validation/ebi-a-GCST90027484.finngen_R12_E4_LACTONAS.pdf]

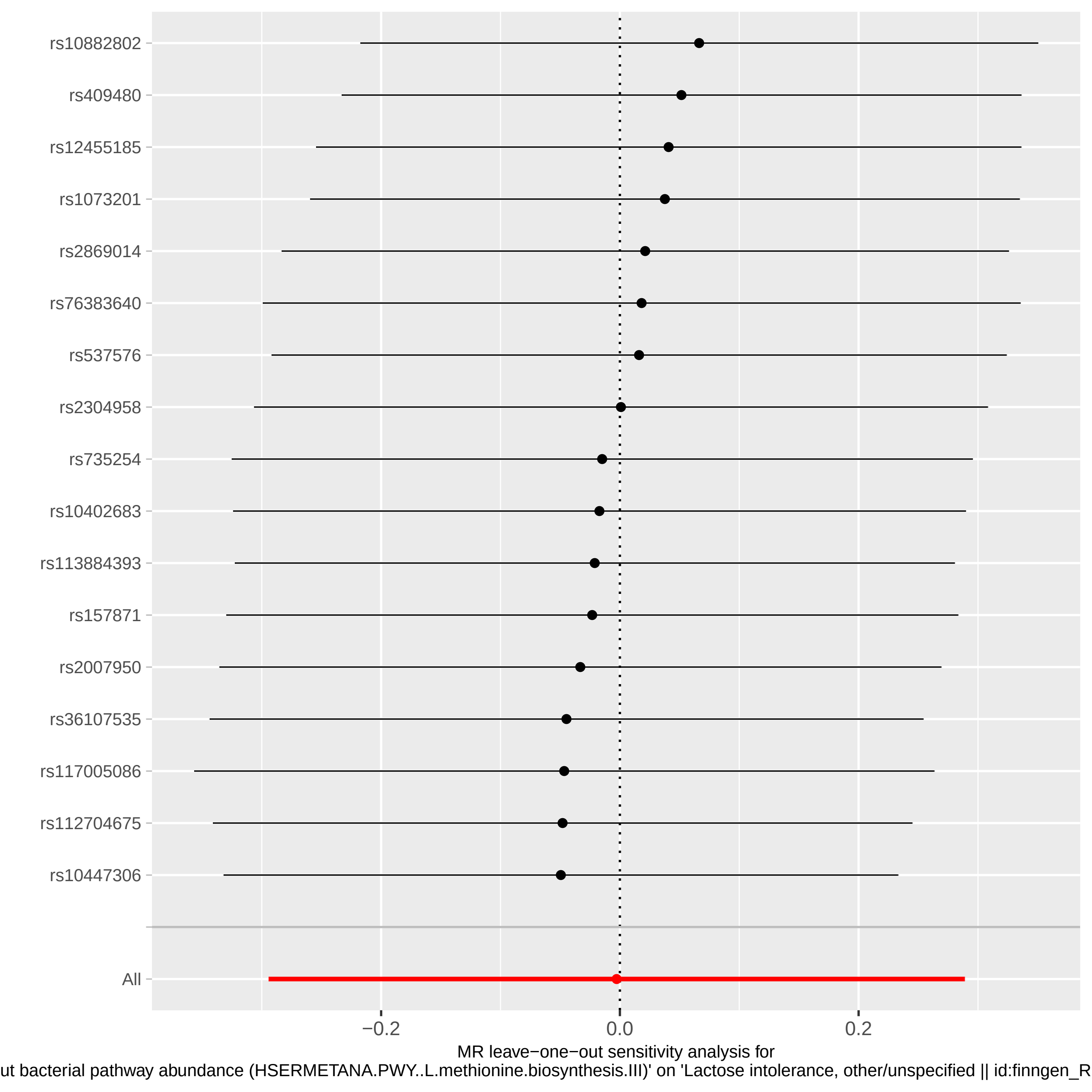
rs10882802
rs409480
rs12455185
rs1073201
rs2869014
rs76383640
rs537576
rs2304958
rs735254
rs10402683
rs113884393
rs157871
rs2007950
rs36107535
rs117005086
rs112704675
rs10447306
All
−0.2
0.0
0.2
MR leave−one−out sensitivity analysis for
ut bacterial pathway abundance (HSERMETANA.PWY..L.methionine.biosynthesis.III)' on 'Lactose intolerance, other/unspecified || id:finngen_R
